# Nodding syndrome, populations at risk need to be aware this is a preventable condition

**DOI:** 10.1093/braincomms/fcad297

**Published:** 2023-10-28

**Authors:** Robert Colebunders, Thomson Lakwo, Amber Hadermann

**Affiliations:** Global Health Institute, University of Antwerp, Antwerp 2610, Belgium; Vector Control Division, Ministry of Health, Kampala, Uganda; Global Health Institute, University of Antwerp, Antwerp 2610, Belgium

We were quite surprised to come across the scientific commentary by Spencer titled ‘New clues to the elusive aetiology of nodding syndrome’.^1^ Upon reading this commentary, we failed to identify any novel insights; instead, we encountered unjustified arguments refuting the potential causative role of *Onchocerca volvulus* in nodding syndrome.

We do not understand why, in the same area in South Sudan, the 2001–02 study results (very strong association between onchocerciasis and nodding syndrome),^2^ and the 2018–19 study results (no association between onchocerciasis and nodding syndrome)^3^ suggest that ‘the nematode infection is a secondary event unrelated to nodding syndrome’.^1^ The difference in results of both case-control studies is explained by (i) the re-introduction in 2017 of a community-directed treatment with ivermectin (CDTI) program decreasing the community microfilarial load and (ii) in the 2018–19 study more persons with nodding syndrome had taken ivermectin compared with controls.^4^ This could mask the association between onchocerciasis and nodding syndrome. Ivermectin intake should be included in the analysis of any research seeking to discover the aetiology of nodding syndrome.

One limitation of case-control studies is that it can be challenging to definitively conclude whether a factor is a real risk factor or merely a consequence of the disease. This is particularly difficult in studies on epilepsy as the life of a person with epilepsy especially in South Sudan will change dramatically once seizures develop.^5^ The longer the time from the onset of seizures, the more the biological findings observed at the time of the study; and this will not represent the clinical status of the person with epilepsy before the onset of the seizures. The median age of persons with nodding syndrome in the 2001–02 study was 12 years (range 5.0–21.0 years)^2^ compared with 15 years (range 9.75–17.0 years) in the more recent study.^3^ Therefore, the duration of disease of persons with nodding syndrome included in the 2018–19 study was much longer, life changes due to the disease and subsequently increasing the and subsequently increasing probability that ‘suspected nodding syndrome risk factors’ were perceived as consequences of the disease.

In the 2018–19 study, three potential new risk factors were identified: (i) reduced viral exposures (ii) *Mansonella perstans* infection and (iii) higher levels of vitamins A and E.^3^ We agree that the role of *M. perstans* as a potential co-risk factor for nodding syndrome in South Sudan needs to be further investigated. However, *M. perstans* cannot be a causal factor in nodding syndrome in certain other regions where nodding syndrome has been reported, such as in Mahenge, Tanzania, where *M. perstans* is not endemic.^6^

In the structural equation model used by Edridge *et al*.,^3^ not only information about the intake of ivermectin and polyvitamins was not available, but also the validity of the model’s outcome is questionable. Indeed, it is difficult to understand that fewer viral exposures would be a risk factor for nodding syndrome. It is more likely that it is a consequence of the disease, given that children with nodding syndrome in South Sudan are generally isolated from their peers and most of them are not allowed to attend school.^4,7^ Consequently, they have fewer viral exposures compared with healthy schoolchildren. The increased vitamin A and E levels are more likely to be related to the intake of polyvitamins because of nodding syndrome-related malnourishment. A link to nodding syndrome exposure to cyanobacteria as suggested by Spencer looks far-fetched and not based on epidemiological evidence.^1^

We noted that the author refers to nodding syndrome as a ‘mysterious disorder’.^1^ In recent years, our understanding of nodding syndrome has significantly advanced. Two cohort studies in Cameroon have demonstrated that it is the level of *O. volvulus* microfilarial load in very young children that is a determining factor for developing epilepsy later in life.^8^ Nodding syndrome, along with other forms of onchocerciasis-associated epilepsy (OAE), occurs in areas where onchocerciasis is poorly controlled or without an onchocerciasis control programme. Affected individuals tend to be clustered in villages and households close to blackfly breeding sites.^9^ Moreover, no new nodding syndrome cases appear when onchocerciasis is successfully eliminated.^10^ In his commentary, Spencer states that water sources could play a causal role in nodding syndrome.^1^ However, this is not because of the use of river water for drinking, cooking, handwashing and bathing but because families are living or farming close to blackfly breeding sites putting their children at risk of being repeatedly bitten by *O. volvulus*-infected blackflies. We agree with Spencer that a causative environmental agent should be able to trigger the tau pathology observed in post-mortem studies of nodding syndrome.^1^ However, it is possible that *O. volvulus* microfilaria, secretory/excretory products or endosymbionts, including viruses, could occasionally cross the weakened blood-brain barrier of young children causing neuroinflammation, resulting in epilepsy and tau deposits.^10^

Evidence-based information about OAE should be disseminated not only to public health policy-makers but also to the affected populations. At the recent 2nd International Workshop on OAE, held from September 19th to 21st in Antwerp, Belgium and organized in collaboration with the WHO Global Onchocerciasis Network for Elimination (GONE) and the WHO Brain Health Unit, recommendations for closer collaboration between the two programmes were proposed. While research concerning the pathogenesis of OAE and the identification of potential causal and protective factors should continue, it was emphasized during this workshop that strengthening of onchocerciasis elimination programmes should be prioritized in areas with high epilepsy prevalence and incidence.^10^ An important part of such a programme should be to increase OAE awareness to motivate populations at risk to take ivermectin in order to prevent children from developing OAE.

## Data Availability

Data sharing is not applicable to this article as no new data were created or analysed.
